# 3,3′-Dibenzyl-1,1′-(2,4,6-trimethyl-*m*-phenyl­enedimethyl­ene)diimidazol-3-ium dibromide

**DOI:** 10.1107/S1600536811005204

**Published:** 2011-02-16

**Authors:** Rosenani A. Haque, Abbas Washeel Salman, Paremala Nadarajan, Madhukar Hemamalini, Hoong-Kun Fun

**Affiliations:** a School of Chemical Sciences, Universiti Sains Malaysia, 11800 USM, Penang, Malaysia; b X-ray Crystallography Unit, School of Physics, Universiti Sains Malaysia, 11800 USM, Penang, Malaysia

## Abstract

In the title molecular salt, C_31_H_34_N_4_^2+^·2Br^−^, the central benzene ring makes dihedral angles of 80.47 (12) and 82.78 (12)° with the adjacent imidazole rings. The dihedral angle between the two terminal phenyl rings is 79.16 (13)°. In the crystal, the cations and anions are linked *via* C—H⋯Br hydrogen bonds, forming supra­molecular chains along the *c* axis.

## Related literature

For applications of *N*-heterocyclic carbenes (NHCs), see: Winkelmann & Navarro (2010 ▶); Papini *et al.* (2008 ▶); Marion *et al.* (2007 ▶); Burstein & Glorius (2004 ▶); Sohn *et al.* (2004 ▶); Grasa *et al.* (2002 ▶); Singh & Nolan (2005 ▶). For the stability of the temperature controller used in the data collection, see: Cosier & Glazer (1986 ▶).
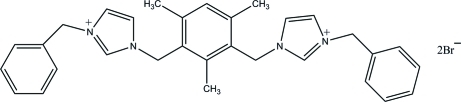


## Experimental

### 

#### Crystal data

C_31_H_34_N_4_^2+^·2Br^−^
*M* *_r_* = 622.44
Monoclinic, 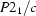

*a* = 8.9851 (2) Å
*b* = 12.8044 (2) Å
*c* = 25.6419 (5) Å
β = 102.611 (1)°
*V* = 2878.90 (10) Å^3^
*Z* = 4
Mo *K*α radiation
μ = 2.84 mm^−1^
*T* = 100 K
0.49 × 0.43 × 0.21 mm

#### Data collection

Bruker SMART APEXII CCD area-detector diffractometer
Absorption correction: multi-scan (*SADABS*; Bruker, 2009 ▶) *T*_min_ = 0.337, *T*_max_ = 0.585
32884 measured reflections
8490 independent reflections
6550 reflections with *I* > 2σ(*I*)
*R*_int_ = 0.036

#### Refinement

*R*[*F* ^2^ > 2σ(*F* ^2^)] = 0.040
*wR*(*F* ^2^) = 0.101
*S* = 1.04
8490 reflections
337 parameters
H-atom parameters constrained
Δρ_max_ = 1.28 e Å^−3^
Δρ_min_ = −0.40 e Å^−3^

### 

Data collection: *APEX2* (Bruker, 2009 ▶); cell refinement: *SAINT* (Bruker, 2009 ▶); data reduction: *SAINT*; program(s) used to solve structure: *SHELXTL* (Sheldrick, 2008 ▶); program(s) used to refine structure: *SHELXTL*; molecular graphics: *SHELXTL*; software used to prepare material for publication: *SHELXTL* and *PLATON* (Spek, 2009 ▶).

## Supplementary Material

Crystal structure: contains datablocks global, I. DOI: 10.1107/S1600536811005204/wn2422sup1.cif

Structure factors: contains datablocks I. DOI: 10.1107/S1600536811005204/wn2422Isup2.hkl

Additional supplementary materials:  crystallographic information; 3D view; checkCIF report

## Figures and Tables

**Table 1:** Hydrogen-bond geometry (Å, °)

*D*—H⋯*A*	*D*—H	H⋯*A*	*D*⋯*A*	*D*—H⋯*A*
C7—H7*A*⋯Br2	0.97	2.90	3.754 (2)	147
C7—H7*B*⋯Br1^i^	0.97	2.92	3.787 (2)	149
C8—H8*A*⋯Br2	0.93	2.81	3.496 (3)	132
C10—H10*A*⋯Br1^i^	0.93	2.74	3.565 (2)	148
C18—H18*B*⋯Br2^ii^	0.97	2.74	3.702 (2)	172
C19—H19*A*⋯Br1^i^	0.93	2.74	3.553 (2)	147
C21—H21*A*⋯Br2^iii^	0.93	2.83	3.603 (3)	141

Symmetry codes: (i) 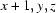; (ii) 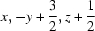; (iii) 
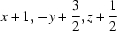
.

**Fig. 1.**
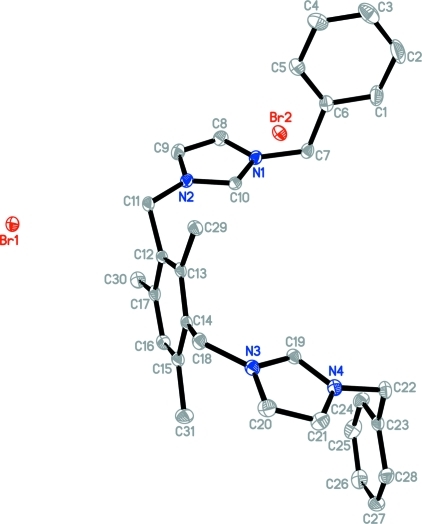
The asymmetric unit of the title compound, showing 30% probability displacement ellipsoids. H atoms have been omitted for clarity.

**Fig. 2.**
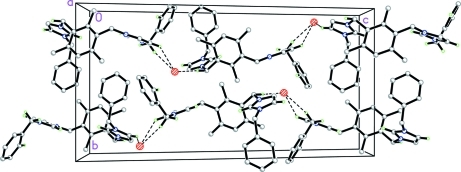
The crystal packing of the title compound, showing hydrogen-bonded (dashed lines) one-dimensional supramolecular chains along the c-axis.

## supplementary crystallographic information

### Comment

*N*-Heterocyclic carbenes (NHCs) have found widespread applications as ligands in organometallic chemistry during recent years (Winkelmann & Navarro, 2010). They typically have strong σ-donor properties but poor π-acceptor character and have been widely employed as alternatives to phosphine ligands to stabilise transition metal complexes.  NHCs are relatively inexpensive, non-toxic and easily prepared from azolium salts (Papini *et al.*, 2008).  Notably, NHCs also exhibit excellent catalytic activity in metal-free organocatalysis (Marion *et al.*, 2007) including umpolung and condensation of carbonyl compounds (Burstein & Glorius, 2004; Sohn *et al.*, 2004) and transesterification reactions (Grasa *et al.*, 2002; Singh & Nolan, 2005).

The asymmetric unit of the title compound, (Fig. 1),  consists of one 1,3-bis(3-benzylimidazolium-1-ylmethyl)mesitylene cation and two bromide anions.   The central benzene ring (C12–C17) makes dihedral angles of  80.47 (12)° and 82.78 (12)° with the adjacent imidazole rings (N1/N2/C8–C10) and (N3/N4/C19–C21). The dihedral angle between the two terminal phenyl rings (C1–C6) and (C23–C28) is 79.16 (13)°.

In the crystal structure (Fig. 2), the cations and anions are linked together *via* intermolecular C—H···Br (Table 1) hydrogen bonds, forming one-dimensional supramolecular chains along the *c*-axis.

### Experimental

A mixture of imidazole (1.0 g, 14.0 mmol) and sodium hydroxide (0.6 g, 15.0 mmol) in DMSO (20 ml) was heated to 363 K for 2 h. The mixture was cooled at room temperature then 1,3-bis(bromomethyl)mesitylene (2.0 g, 6.5 mmol) in 10 ml of DMSO was added, heated to 413 K for 1 h and poured into water (200 ml), then cooled in an ice bath. The resulting precipitate was collected by filtration, washed with water (3x10 ml), and recrystallised from methanol/water to give 1,3-bis(*N*-imidazole-1-ylmethyl)mesitylene as an off-white solid (1.45 g, 79 %). Further, a mixture of 1,3-bis(*N*-imidazole-1-ylmethyl)mesitylene (0.7 g, 2.5 mmol) and benzyl bromide (1.0 g, 5.8 mmol) in 30 ml of acetonitrile, was refluxed for 24 h, then cooled to room temperature and left standing overnight, giving the title compound as light brown crystals which were isolated by decantation and washed with diethyl ether (2x5 ml) and placed in a desiccator. The yield was  (1.15 g, 74%). The resulting crystals were suitable for X-ray diffraction.

### Refinement

All H atoms were positioned geometrically [C–H = 0.93–0.97 Å] and were refined using a riding model, with *U*_iso_(H) = x*U*_eq_(C), where x = 1.5 for methyl H and 1.2 for all other H atoms. The highest peak in the final difference map was found at a distance of 0.77 Å from Br1.

### Crystal data

**Table:**

C_31_H_34_N_4_^2+^·2Br^−^	*F*(000) = 1272
*M**_r_* = 622.44	*D*_x_ = 1.436 Mg m^−^^3^
Monoclinic, *P*2_1_/*c*	Mo *K*α radiation, λ = 0.71073 Å
Hall symbol: -P 2ybc	Cell parameters from 9955 reflections
*a* = 8.9851 (2) Å	θ = 2.3–29.9°
*b* = 12.8044 (2) Å	µ = 2.84 mm^−^^1^
*c* = 25.6419 (5) Å	*T* = 100 K
β = 102.611 (1)°	Plate, colourless
*V* = 2878.90 (10) Å^3^	0.49 × 0.43 × 0.21 mm
*Z* = 4	

### Data collection

**Table:**

Bruker SMART APEXII CCD area-detector diffractometer	8490 independent reflections
Radiation source: fine-focus sealed tube	6550 reflections with *I* > 2σ(*I*)
graphite	*R*_int_ = 0.036
φ and ω scans	θ_max_ = 30.2°, θ_min_ = 2.3°
Absorption correction: multi-scan (*SADABS*; Bruker, 2009)	*h* = −12→12
*T*_min_ = 0.337, *T*_max_ = 0.585	*k* = −17→18
32884 measured reflections	*l* = −30→36

### Refinement

**Table:**

Refinement on *F*^2^	Primary atom site location: structure-invariant direct methods
Least-squares matrix: full	Secondary atom site location: difference Fourier map
*R*[*F*^2^ > 2σ(*F*^2^)] = 0.040	Hydrogen site location: inferred from neighbouring sites
*wR*(*F*^2^) = 0.101	H-atom parameters constrained
*S* = 1.04	*w* = 1/[σ^2^(*F*_o_^2^) + (0.0549*P*)^2^ + 0.6972*P*] where *P* = (*F*_o_^2^ + 2*F*_c_^2^)/3
8490 reflections	(Δ/σ)_max_ = 0.003
337 parameters	Δρ_max_ = 1.28 e Å^−^^3^
0 restraints	Δρ_min_ = −0.40 e Å^−^^3^

### Special details

**Table:**

Experimental. The crystal was placed in the cold stream of an Oxford Cryosystems Cobra open-flow nitrogen cryostat (Cosier & Glazer, 1986) operating at 100.0 (1) K.
Geometry. All s.u.'s (except the s.u. in the dihedral angle between two l.s. planes) are estimated using the full covariance matrix. The cell s.u.'s are taken into account individually in the estimation of s.u.'s in distances, angles and torsion angles; correlations between s.u.'s in cell parameters are only used when they are defined by crystal symmetry. An approximate (isotropic) treatment of cell s.u.'s is used for estimating s.u.'s involving l.s. planes.
Refinement. Refinement of F^2^ against ALL reflections. The weighted R-factor wR and goodness of fit S are based on F^2^, conventional R-factors R are based on F, with F set to zero for negative F^2^. The threshold expression of F^2^ > 2σ(F^2^) is used only for calculating R-factors(gt) etc. and is not relevant to the choice of reflections for refinement. R-factors based on F^2^ are statistically about twice as large as those based on F, and R- factors based on ALL data will be even larger.

### Fractional atomic coordinates and isotropic or equivalent isotropic displacement parameters (Å^2^)

**Table:**

	*x*	*y*	*z*	*U*_iso_*/*U*_eq_	
N1	0.6565 (2)	0.78480 (15)	0.80723 (7)	0.0240 (4)	
N2	0.4998 (2)	0.82215 (14)	0.85747 (8)	0.0235 (4)	
N3	0.9596 (2)	0.88648 (15)	1.09775 (8)	0.0244 (4)	
N4	1.1823 (2)	0.82656 (14)	1.09554 (8)	0.0242 (4)	
C1	0.9744 (3)	0.8645 (3)	0.74204 (12)	0.0452 (7)	
H1A	1.0412	0.8088	0.7517	0.054*	
C2	1.0150 (4)	0.9467 (3)	0.71276 (14)	0.0591 (9)	
H2A	1.1091	0.9461	0.7033	0.071*	
C3	0.9160 (4)	1.0297 (3)	0.69757 (12)	0.0504 (8)	
H3A	0.9425	1.0845	0.6776	0.060*	
C4	0.7775 (3)	1.0297 (2)	0.71252 (11)	0.0395 (6)	
H4A	0.7102	1.0850	0.7024	0.047*	
C5	0.7372 (3)	0.94820 (19)	0.74249 (10)	0.0309 (5)	
H5A	0.6441	0.9499	0.7527	0.037*	
C6	0.8349 (3)	0.86441 (19)	0.75719 (9)	0.0274 (5)	
C7	0.7989 (3)	0.77211 (19)	0.78865 (9)	0.0268 (5)	
H7A	0.7912	0.7101	0.7665	0.032*	
H7B	0.8822	0.7616	0.8193	0.032*	
C8	0.5118 (3)	0.76704 (19)	0.77738 (10)	0.0292 (5)	
H8A	0.4861	0.7428	0.7424	0.035*	
C9	0.4139 (3)	0.79170 (19)	0.80890 (10)	0.0298 (5)	
H9A	0.3080	0.7886	0.7993	0.036*	
C10	0.6473 (2)	0.81899 (16)	0.85554 (9)	0.0216 (4)	
H10A	0.7289	0.8374	0.8830	0.026*	
C11	0.4391 (2)	0.85726 (18)	0.90348 (9)	0.0248 (4)	
H11A	0.3343	0.8340	0.8989	0.030*	
H11B	0.4393	0.9330	0.9046	0.030*	
C12	0.5310 (2)	0.81582 (16)	0.95579 (9)	0.0226 (4)	
C13	0.6366 (2)	0.87950 (16)	0.98984 (9)	0.0225 (4)	
C14	0.7047 (2)	0.84395 (17)	1.04123 (9)	0.0236 (4)	
C15	0.6774 (3)	0.74111 (18)	1.05665 (9)	0.0261 (5)	
C16	0.5809 (3)	0.67784 (17)	1.02034 (10)	0.0272 (5)	
H16A	0.5668	0.6090	1.0298	0.033*	
C17	0.5045 (2)	0.71272 (17)	0.97056 (9)	0.0249 (5)	
C18	0.7980 (3)	0.91657 (17)	1.08167 (10)	0.0269 (5)	
H18A	0.7914	0.9865	1.0668	0.032*	
H18B	0.7547	0.9183	1.1132	0.032*	
C19	1.0401 (2)	0.83681 (17)	1.06739 (9)	0.0236 (4)	
H19A	1.0032	0.8134	1.0326	0.028*	
C20	1.0534 (3)	0.90914 (19)	1.14621 (10)	0.0322 (5)	
H20A	1.0258	0.9440	1.1745	0.039*	
C21	1.1933 (3)	0.87145 (19)	1.14526 (10)	0.0315 (5)	
H21A	1.2800	0.8750	1.1727	0.038*	
C22	1.3042 (3)	0.76808 (17)	1.07770 (10)	0.0268 (5)	
H22A	1.4030	0.7916	1.0976	0.032*	
H22B	1.2985	0.7816	1.0401	0.032*	
C23	1.2878 (2)	0.65205 (18)	1.08634 (9)	0.0236 (4)	
C24	1.1996 (3)	0.59206 (19)	1.04611 (10)	0.0307 (5)	
H24A	1.1554	0.6224	1.0134	0.037*	
C25	1.1774 (3)	0.4869 (2)	1.05463 (12)	0.0395 (6)	
H25A	1.1199	0.4464	1.0274	0.047*	
C26	1.2404 (3)	0.44214 (19)	1.10336 (12)	0.0385 (6)	
H26A	1.2218	0.3723	1.1095	0.046*	
C27	1.3312 (3)	0.5008 (2)	1.14307 (11)	0.0360 (6)	
H27A	1.3760	0.4701	1.1756	0.043*	
C28	1.3553 (3)	0.6056 (2)	1.13442 (10)	0.0304 (5)	
H28A	1.4171	0.6450	1.1611	0.036*	
C29	0.6784 (3)	0.98590 (17)	0.97157 (10)	0.0263 (5)	
H29A	0.7857	0.9975	0.9843	0.039*	
H29B	0.6224	1.0388	0.9856	0.039*	
H29C	0.6535	0.9888	0.9332	0.039*	
C30	0.3955 (3)	0.64136 (19)	0.93407 (11)	0.0315 (5)	
H30A	0.3917	0.5752	0.9513	0.047*	
H30B	0.4297	0.6314	0.9015	0.047*	
H30C	0.2956	0.6721	0.9262	0.047*	
C31	0.7495 (3)	0.69928 (19)	1.11128 (10)	0.0336 (5)	
H31A	0.7026	0.6341	1.1168	0.050*	
H31B	0.7352	0.7485	1.1380	0.050*	
H31C	0.8566	0.6887	1.1138	0.050*	
Br1	0.03325 (2)	0.815324 (18)	0.928724 (9)	0.02679 (7)	
Br2	0.59309 (3)	0.572410 (19)	0.692096 (9)	0.03010 (7)	

### Atomic displacement parameters (Å^2^)

**Table:**

	*U*^11^	*U*^22^	*U*^33^	*U*^12^	*U*^13^	*U*^23^
N1	0.0227 (9)	0.0246 (9)	0.0250 (9)	0.0029 (7)	0.0060 (7)	−0.0007 (7)
N2	0.0216 (9)	0.0207 (9)	0.0288 (10)	0.0030 (7)	0.0070 (7)	0.0024 (7)
N3	0.0271 (9)	0.0217 (9)	0.0264 (10)	0.0038 (7)	0.0100 (7)	0.0010 (8)
N4	0.0245 (9)	0.0191 (9)	0.0306 (10)	0.0010 (7)	0.0095 (7)	0.0012 (7)
C1	0.0230 (12)	0.066 (2)	0.0476 (16)	−0.0013 (13)	0.0093 (11)	0.0009 (15)
C2	0.0332 (15)	0.089 (3)	0.060 (2)	−0.0205 (16)	0.0208 (14)	0.0009 (19)
C3	0.0549 (19)	0.0547 (19)	0.0431 (17)	−0.0281 (16)	0.0142 (14)	−0.0028 (14)
C4	0.0529 (17)	0.0309 (14)	0.0354 (14)	−0.0077 (12)	0.0111 (12)	−0.0017 (11)
C5	0.0350 (13)	0.0304 (13)	0.0296 (12)	−0.0032 (10)	0.0118 (10)	−0.0056 (10)
C6	0.0244 (11)	0.0336 (13)	0.0242 (11)	−0.0037 (9)	0.0050 (9)	−0.0073 (9)
C7	0.0245 (11)	0.0302 (12)	0.0265 (11)	0.0061 (9)	0.0071 (9)	−0.0041 (9)
C8	0.0279 (11)	0.0306 (13)	0.0274 (12)	−0.0010 (9)	0.0025 (9)	−0.0031 (10)
C9	0.0224 (11)	0.0298 (12)	0.0361 (13)	−0.0006 (9)	0.0038 (9)	−0.0004 (10)
C10	0.0224 (10)	0.0192 (10)	0.0234 (10)	0.0019 (8)	0.0055 (8)	0.0009 (8)
C11	0.0228 (10)	0.0236 (11)	0.0307 (12)	0.0053 (8)	0.0119 (9)	0.0026 (9)
C12	0.0235 (10)	0.0201 (10)	0.0281 (11)	0.0066 (8)	0.0140 (8)	0.0039 (9)
C13	0.0243 (10)	0.0171 (10)	0.0311 (12)	0.0048 (8)	0.0167 (9)	0.0025 (8)
C14	0.0226 (10)	0.0220 (11)	0.0304 (12)	0.0052 (8)	0.0149 (9)	0.0035 (9)
C15	0.0272 (11)	0.0234 (11)	0.0316 (12)	0.0078 (9)	0.0152 (9)	0.0069 (9)
C16	0.0326 (12)	0.0173 (11)	0.0361 (13)	0.0053 (9)	0.0171 (10)	0.0052 (9)
C17	0.0248 (10)	0.0186 (10)	0.0356 (12)	0.0029 (8)	0.0160 (9)	0.0003 (9)
C18	0.0278 (11)	0.0222 (11)	0.0339 (12)	0.0064 (9)	0.0136 (9)	0.0017 (9)
C19	0.0256 (11)	0.0205 (11)	0.0267 (11)	0.0010 (8)	0.0102 (9)	0.0015 (8)
C20	0.0388 (13)	0.0311 (13)	0.0274 (12)	0.0054 (10)	0.0089 (10)	−0.0037 (10)
C21	0.0336 (13)	0.0279 (13)	0.0314 (13)	0.0051 (10)	0.0032 (10)	−0.0044 (10)
C22	0.0230 (10)	0.0221 (11)	0.0386 (13)	0.0011 (8)	0.0138 (9)	0.0012 (9)
C23	0.0212 (10)	0.0224 (11)	0.0296 (11)	0.0034 (8)	0.0106 (8)	0.0024 (9)
C24	0.0291 (12)	0.0268 (12)	0.0345 (13)	0.0019 (9)	0.0031 (10)	0.0031 (10)
C25	0.0333 (13)	0.0279 (13)	0.0540 (17)	−0.0006 (10)	0.0022 (12)	−0.0048 (12)
C26	0.0368 (14)	0.0186 (12)	0.0631 (19)	0.0061 (10)	0.0175 (13)	0.0087 (11)
C27	0.0382 (14)	0.0359 (14)	0.0366 (14)	0.0139 (11)	0.0142 (11)	0.0137 (11)
C28	0.0283 (12)	0.0329 (13)	0.0304 (12)	0.0089 (10)	0.0074 (9)	−0.0010 (10)
C29	0.0306 (11)	0.0195 (11)	0.0322 (12)	0.0034 (9)	0.0143 (9)	0.0045 (9)
C30	0.0342 (13)	0.0211 (11)	0.0415 (14)	0.0000 (10)	0.0135 (11)	−0.0001 (10)
C31	0.0408 (14)	0.0254 (12)	0.0356 (14)	0.0047 (10)	0.0100 (11)	0.0089 (10)
Br1	0.02077 (11)	0.03140 (13)	0.02841 (12)	0.00005 (9)	0.00584 (8)	0.00150 (9)
Br2	0.03523 (13)	0.02893 (13)	0.02839 (13)	−0.00436 (9)	0.01185 (9)	−0.00585 (9)

### Geometric parameters (Å, °)

**Table:**

N1—C10	1.333 (3)	C14—C15	1.412 (3)
N1—C8	1.376 (3)	C14—C18	1.504 (3)
N1—C7	1.468 (3)	C15—C16	1.387 (4)
N2—C10	1.337 (3)	C15—C31	1.507 (3)
N2—C9	1.371 (3)	C16—C17	1.385 (3)
N2—C11	1.474 (3)	C16—H16A	0.9300
N3—C19	1.334 (3)	C17—C30	1.506 (3)
N3—C20	1.371 (3)	C18—H18A	0.9700
N3—C18	1.472 (3)	C18—H18B	0.9700
N4—C19	1.330 (3)	C19—H19A	0.9300
N4—C21	1.382 (3)	C20—C21	1.352 (3)
N4—C22	1.479 (3)	C20—H20A	0.9300
C1—C2	1.387 (4)	C21—H21A	0.9300
C1—C6	1.391 (3)	C22—C23	1.514 (3)
C1—H1A	0.9300	C22—H22A	0.9700
C2—C3	1.386 (5)	C22—H22B	0.9700
C2—H2A	0.9300	C23—C28	1.384 (3)
C3—C4	1.380 (4)	C23—C24	1.388 (3)
C3—H3A	0.9300	C24—C25	1.385 (3)
C4—C5	1.390 (4)	C24—H24A	0.9300
C4—H4A	0.9300	C25—C26	1.379 (4)
C5—C6	1.385 (3)	C25—H25A	0.9300
C5—H5A	0.9300	C26—C27	1.380 (4)
C6—C7	1.505 (3)	C26—H26A	0.9300
C7—H7A	0.9700	C27—C28	1.384 (4)
C7—H7B	0.9700	C27—H27A	0.9300
C8—C9	1.355 (3)	C28—H28A	0.9300
C8—H8A	0.9300	C29—H29A	0.9600
C9—H9A	0.9300	C29—H29B	0.9600
C10—H10A	0.9300	C29—H29C	0.9600
C11—C12	1.510 (3)	C30—H30A	0.9600
C11—H11A	0.9700	C30—H30B	0.9600
C11—H11B	0.9700	C30—H30C	0.9600
C12—C13	1.402 (3)	C31—H31A	0.9600
C12—C17	1.408 (3)	C31—H31B	0.9600
C13—C14	1.402 (3)	C31—H31C	0.9600
C13—C29	1.515 (3)		
			
C10—N1—C8	109.14 (18)	C17—C16—C15	122.8 (2)
C10—N1—C7	124.97 (19)	C17—C16—H16A	118.6
C8—N1—C7	125.85 (19)	C15—C16—H16A	118.6
C10—N2—C9	108.90 (18)	C16—C17—C12	118.1 (2)
C10—N2—C11	125.60 (19)	C16—C17—C30	120.1 (2)
C9—N2—C11	125.44 (19)	C12—C17—C30	121.8 (2)
C19—N3—C20	109.00 (19)	N3—C18—C14	113.49 (18)
C19—N3—C18	126.1 (2)	N3—C18—H18A	108.9
C20—N3—C18	124.88 (19)	C14—C18—H18A	108.9
C19—N4—C21	109.05 (19)	N3—C18—H18B	108.9
C19—N4—C22	124.8 (2)	C14—C18—H18B	108.9
C21—N4—C22	125.9 (2)	H18A—C18—H18B	107.7
C2—C1—C6	120.8 (3)	N4—C19—N3	108.0 (2)
C2—C1—H1A	119.6	N4—C19—H19A	126.0
C6—C1—H1A	119.6	N3—C19—H19A	126.0
C3—C2—C1	120.4 (3)	C21—C20—N3	107.3 (2)
C3—C2—H2A	119.8	C21—C20—H20A	126.3
C1—C2—H2A	119.8	N3—C20—H20A	126.3
C4—C3—C2	119.0 (3)	C20—C21—N4	106.6 (2)
C4—C3—H3A	120.5	C20—C21—H21A	126.7
C2—C3—H3A	120.5	N4—C21—H21A	126.7
C3—C4—C5	120.9 (3)	N4—C22—C23	110.43 (17)
C3—C4—H4A	119.6	N4—C22—H22A	109.6
C5—C4—H4A	119.6	C23—C22—H22A	109.6
C6—C5—C4	120.4 (2)	N4—C22—H22B	109.6
C6—C5—H5A	119.8	C23—C22—H22B	109.6
C4—C5—H5A	119.8	H22A—C22—H22B	108.1
C5—C6—C1	118.7 (2)	C28—C23—C24	119.5 (2)
C5—C6—C7	123.8 (2)	C28—C23—C22	121.0 (2)
C1—C6—C7	117.5 (2)	C24—C23—C22	119.5 (2)
N1—C7—C6	113.04 (19)	C25—C24—C23	119.9 (2)
N1—C7—H7A	109.0	C25—C24—H24A	120.0
C6—C7—H7A	109.0	C23—C24—H24A	120.0
N1—C7—H7B	109.0	C26—C25—C24	120.2 (3)
C6—C7—H7B	109.0	C26—C25—H25A	119.9
H7A—C7—H7B	107.8	C24—C25—H25A	119.9
C9—C8—N1	106.7 (2)	C25—C26—C27	120.1 (2)
C9—C8—H8A	126.6	C25—C26—H26A	119.9
N1—C8—H8A	126.6	C27—C26—H26A	119.9
C8—C9—N2	107.3 (2)	C26—C27—C28	119.8 (2)
C8—C9—H9A	126.3	C26—C27—H27A	120.1
N2—C9—H9A	126.3	C28—C27—H27A	120.1
N1—C10—N2	107.92 (19)	C23—C28—C27	120.4 (2)
N1—C10—H10A	126.0	C23—C28—H28A	119.8
N2—C10—H10A	126.0	C27—C28—H28A	119.8
N2—C11—C12	112.17 (17)	C13—C29—H29A	109.5
N2—C11—H11A	109.2	C13—C29—H29B	109.5
C12—C11—H11A	109.2	H29A—C29—H29B	109.5
N2—C11—H11B	109.2	C13—C29—H29C	109.5
C12—C11—H11B	109.2	H29A—C29—H29C	109.5
H11A—C11—H11B	107.9	H29B—C29—H29C	109.5
C13—C12—C17	120.6 (2)	C17—C30—H30A	109.5
C13—C12—C11	120.90 (19)	C17—C30—H30B	109.5
C17—C12—C11	118.5 (2)	H30A—C30—H30B	109.5
C12—C13—C14	119.6 (2)	C17—C30—H30C	109.5
C12—C13—C29	120.7 (2)	H30A—C30—H30C	109.5
C14—C13—C29	119.7 (2)	H30B—C30—H30C	109.5
C13—C14—C15	119.9 (2)	C15—C31—H31A	109.5
C13—C14—C18	120.7 (2)	C15—C31—H31B	109.5
C15—C14—C18	119.2 (2)	H31A—C31—H31B	109.5
C16—C15—C14	118.6 (2)	C15—C31—H31C	109.5
C16—C15—C31	119.7 (2)	H31A—C31—H31C	109.5
C14—C15—C31	121.7 (2)	H31B—C31—H31C	109.5
			
C6—C1—C2—C3	−0.7 (5)	C13—C14—C15—C31	179.4 (2)
C1—C2—C3—C4	0.7 (5)	C18—C14—C15—C31	−5.0 (3)
C2—C3—C4—C5	0.2 (4)	C14—C15—C16—C17	−3.1 (3)
C3—C4—C5—C6	−1.0 (4)	C31—C15—C16—C17	176.5 (2)
C4—C5—C6—C1	0.9 (4)	C15—C16—C17—C12	2.4 (3)
C4—C5—C6—C7	−178.8 (2)	C15—C16—C17—C30	−177.1 (2)
C2—C1—C6—C5	−0.1 (4)	C13—C12—C17—C16	2.5 (3)
C2—C1—C6—C7	179.7 (3)	C11—C12—C17—C16	−175.25 (18)
C10—N1—C7—C6	−96.1 (3)	C13—C12—C17—C30	−178.02 (19)
C8—N1—C7—C6	81.5 (3)	C11—C12—C17—C30	4.2 (3)
C5—C6—C7—N1	−6.2 (3)	C19—N3—C18—C14	30.3 (3)
C1—C6—C7—N1	174.1 (2)	C20—N3—C18—C14	−152.4 (2)
C10—N1—C8—C9	0.2 (3)	C13—C14—C18—N3	−116.1 (2)
C7—N1—C8—C9	−177.8 (2)	C15—C14—C18—N3	68.3 (3)
N1—C8—C9—N2	−1.0 (3)	C21—N4—C19—N3	−0.3 (3)
C10—N2—C9—C8	1.6 (3)	C22—N4—C19—N3	174.75 (19)
C11—N2—C9—C8	179.0 (2)	C20—N3—C19—N4	0.5 (3)
C8—N1—C10—N2	0.8 (2)	C18—N3—C19—N4	178.19 (19)
C7—N1—C10—N2	178.76 (19)	C19—N3—C20—C21	−0.6 (3)
C9—N2—C10—N1	−1.5 (2)	C18—N3—C20—C21	−178.2 (2)
C11—N2—C10—N1	−178.90 (19)	N3—C20—C21—N4	0.4 (3)
C10—N2—C11—C12	−42.9 (3)	C19—N4—C21—C20	0.0 (3)
C9—N2—C11—C12	140.1 (2)	C22—N4—C21—C20	−175.0 (2)
N2—C11—C12—C13	101.9 (2)	C19—N4—C22—C23	−79.9 (3)
N2—C11—C12—C17	−80.3 (2)	C21—N4—C22—C23	94.4 (3)
C17—C12—C13—C14	−6.5 (3)	N4—C22—C23—C28	−88.0 (3)
C11—C12—C13—C14	171.21 (18)	N4—C22—C23—C24	89.8 (3)
C17—C12—C13—C29	173.11 (18)	C28—C23—C24—C25	1.2 (3)
C11—C12—C13—C29	−9.2 (3)	C22—C23—C24—C25	−176.7 (2)
C12—C13—C14—C15	5.7 (3)	C23—C24—C25—C26	1.2 (4)
C29—C13—C14—C15	−173.94 (18)	C24—C25—C26—C27	−2.7 (4)
C12—C13—C14—C18	−169.92 (18)	C25—C26—C27—C28	1.8 (4)
C29—C13—C14—C18	10.4 (3)	C24—C23—C28—C27	−2.1 (3)
C13—C14—C15—C16	−1.0 (3)	C22—C23—C28—C27	175.7 (2)
C18—C14—C15—C16	174.71 (19)	C26—C27—C28—C23	0.6 (4)

### Hydrogen-bond geometry (Å, °)

**Table:**

*D*—H···*A*	*D*—H	H···*A*	*D*···*A*	*D*—H···*A*
C7—H7A···Br2	0.97	2.90	3.754 (2)	147
C7—H7B···Br1^i^	0.97	2.92	3.787 (2)	149
C8—H8A···Br2	0.93	2.81	3.496 (3)	132
C10—H10A···Br1^i^	0.93	2.74	3.565 (2)	148
C18—H18B···Br2^ii^	0.97	2.74	3.702 (2)	172
C19—H19A···Br1^i^	0.93	2.74	3.553 (2)	147
C21—H21A···Br2^iii^	0.93	2.83	3.603 (3)	141

Symmetry codes: (i) *x*+1, *y*, *z*; (ii) *x*, −*y*+3/2, *z*+1/2; (iii) *x*+1, −*y*+3/2, *z*+1/2.

## References

[bb1] Bruker (2009). *APEX2*, *SAINT* and *SADABS* Bruker AXS Inc., Madison, Wisconsin, USA.

[bb2] Burstein, C. & Glorius, F. (2004). *Angew. Chem. Int. Ed.* **43**, 6205–6208. 10.1002/anie.200461572 15549739

[bb3] Cosier, J. & Glazer, A. M. (1986). *J. Appl. Cryst.* **19**, 105–107.

[bb4] Grasa, G. A., Kissling, R. M. & Nolan, S. P. (2002). *Org. Lett.* **4**, 3583–3586. 10.1021/ol0264760 12375893

[bb5] Marion, N., Diez-Gonzáles, S. & Nolan, S. P. (2007). *Angew. Chem. Int. Ed.* **46**, 2988–3000. 10.1002/anie.200603380 17348057

[bb6] Papini, G., Bandoli, G., Dolmella, A., Lobbia, G. G., Pellei, M. & Santini, C. (2008). *Inorg. Chem. Commun.* **11**, 1103–1106.

[bb7] Sheldrick, G. M. (2008). *Acta Cryst.* A**64**, 112–122. 10.1107/S0108767307043930 18156677

[bb8] Singh, R. & Nolan, S. P. (2005). *Chem. Commun.* pp. 5456–5458. 10.1039/b509783e 16261245

[bb9] Sohn, S. S., Rosen, E. L. & Bode, J. W. (2004). *J. Am. Chem. Soc.* **126**, 14370–14371. 10.1021/ja044714b 15521753

[bb10] Spek, A. L. (2009). *Acta Cryst.* D**65**, 148–155. 10.1107/S090744490804362X PMC2631630 19171970

[bb11] Winkelmann, O. H. & Navarro, O. (2010). *Adv. Synth. Catal.* **352**, 212–214.

